# Ceramsite Facilitated Microbial Degradation of Pollutants in Domestic Wastewater

**DOI:** 10.3390/ijerph17134692

**Published:** 2020-06-30

**Authors:** Qiong Wan, Qingji Han, Hailin Luo, Tao He, Feng Xue, Zihuizhong Ye, Chen Chen, Shan Huang

**Affiliations:** 1 School of Architecture and Civil Engineering, Xi’an University of Science and Technology, Xi’an 710054, China; wq6675@xust.edu.cn; 2 Xi’an Research and Design Institute of Wall & Roof Materials Co., Ltd., Xi’an 710061, China; hanqingjih@163.com; 3 State Environmental Protection Key Laboratory of Urban Ecological Environment Simulation and Protection, South China Institute of Environmental Sciences, Ministry of Ecology and Environment of China, Guangzhou 510535, China; luohailin@scies.org (H.L.); hetao@scies.org (T.H.); 4 Xi’an Pengyi Environmental Engineering co. Ltd., Xi’an 710054, China; melon75@163.com; 5 Stuart Country Day School, Princeton, NJ 08540, USA; zye_22@stuartschool.org; 6 Department of Civil and Environmental Engineering, Princeton University, Princeton, NJ 08544, USA; shanh@princeton.edu

**Keywords:** constructed wetland, ceramsite substrate, microbial community component, nitrogen functional microorganisms

## Abstract

Although constructed wetlands (CWs) are widely used around the world with various substrates, the mechanisms of how these modified substrates affect wastewater treatment are still unknown. In this study, CW microcosms were established with and without ceramsite as a substrate, and the wastewater treatment efficiencies were evaluated during 71 days of incubation. Using the 16S rRNA high-through sequencing, the mechanisms of how CW substrate changed the microbial community was quantified. The results showed that compared to soil as substrate, the use of ceramsite as substrate material enhanced the removal of pollutants from CW systems, particularly under a short retention time (1.5-day) condition. There were more beneficial microorganism groups (nitrogen, sulfur, phosphate) in the ceramsite CW system than the non-ceramsite CW system, particularly in the bottom layers. Moreover, the CW with ceramsite substrate had more nitrification function. All of these results suggested that the ceramsite CW system enhanced the removal of pollutants because it increased the concentration of key microbes that are necessarily for nutrient cycles.

## 1. Introduction

Overloading nitrogen (N) will cause a deterioration of water quality and lead to the eutrophication of aquatic ecosystems [1,2]. Therefore, it is necessary to reduce N as well as other pollutants from domestic and industrial wastewater before discharging it into natural water bodies. Compared to wastewater treatment plants and anaerobic digester reactors, constructed wetlands (CWs) are more economically and environmentally friendly. CW systems are widely used to treat nonpoint source wastewater in areas that lack wastewater collecting systems [3,4,5].

CWs are comprehensive systems that remove pollutants (organic compounds, N, and phosphorus (P) via various processes, including sedimentation, filtration, volatilization, plant uptake and microbial degradation) [5,6]. Among them, it is well recognized that the removal of nutrient pollutants is primarily due to microbial degradation [7,8,9]. Therefore, strengthening the functional microbes can thus enhance pollutant removal efficiency of the CWs. In particular, organic compounds can be degraded and mineralized by both aerobic and anaerobic microbes through various oxidation-reduction reactions [10,11]. N removal is usually a combination of nitrification and denitrification, sometimes as well as the anammox process [12,13]. Many microorganisms that are involved in sulfur (S) and P reactions also contribute to heavy metal removal [14,15,16]. Thus, to further enhance the efficiency of pollutant removal in CWs, we need a better understanding of the microbial communities in this system to promote the activity of specific functional groups.

Another feasible way to improve wastewater treatment efficiency is to select suitable substrate materials [17,18,19]. CW substrates are important as they support the growth of wetland plants and provide a good habitat for the development of biofilms of microorganisms. The suitable substrate should be easily to obtain, are cheap, satisfy the hydraulic and engineering feasibility, and have few secondary pollution issues [6,20]. Recently, ceramsite, which has high porosity to provide more space for the settlement of plants and maintain aerobic conditions, has been considered a potential CW substrate material [21,22]. Additionally, with the high absorption capability, ceramsite is able to gather nutrient and pollutants thus providing sufficient substrates for microbial degradation. Meanwhile, ceramsite can be made of old sludge from wastewater treatment; therefore, the manufacture of this material is low cost and conducive to resource recycle.

It is promising to use ceramsite substrates to simulate the microbial pollutant degradation ability to improve the efficacy of wastewater treatment in CWs. However, the mechanisms of ceramsite motivating microbial activity require more in-depth research before it can be widely apply to CW wastewater treatment systems. In this case, the aims of this study were to compare the wastewater treatment efficiency between ceramsite and soil in CW systems, and discover the key microbial community for pollutant removal under different conditions.

## 2. Materials and Methods

### 2.1. Preparation of Ceramsite from Sludge

Sludge was collected from a water treatment plant in Xi’an city, China. The procedure to prepare ceramsite are described as follows: Mixtures with 60% sludge, 16% coal flying ash, 16% clay and 8% glass power were first incubated at 500 °C for 20 min, increased by 10 °C per minute to reach a temperature of 1170 °C, and then maintain them at 1170 °C for 20 min. Ceramsite used as wetland substrates had an apparent density of 1.388 g/cm^3^, bulk density of 0.764 g/cm^3^, specific surface area of 5.24 × 10^4^ cm^2^/g, porosity of 45.0% and a water absorption capacity of 23.65%. The leaching rate of heavy metals (barium, arsenic, zinc, nickel, lead, chromium, copper, cadmium) was tested. All of the leaching concentrations of heavy metals were less than 1.68 mg/L, under limited quality controlled conditions (Table S1).

### 2.2. Set up of Wetland Microcosms

Wetland soils were collected from Nansha Wetland Park (22°37’04’’ N, 113°38’23’’ E), located in Guangzhou City that was near Pearl River Estuary, China. *Bruguiera gymnorhiza*, a typical mangrove plant in the wetland of the Pearl River Estuary areas, was also collected at the same time.

**Figure 1:**
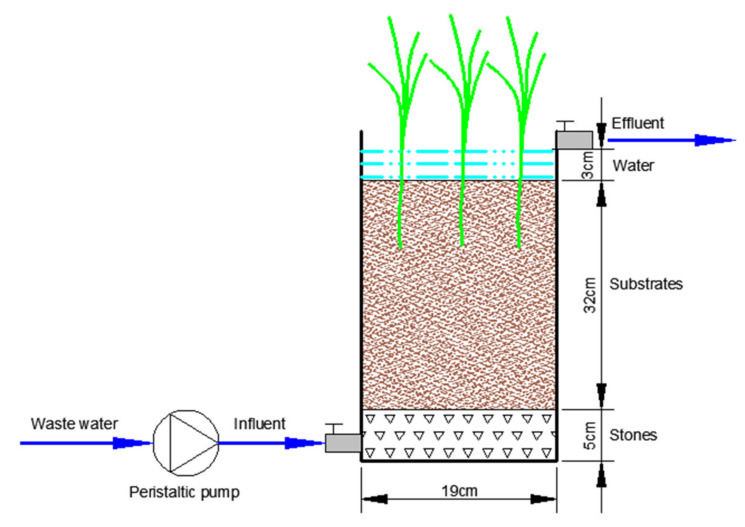
The schematic diagram of the experimental constructed wetland system.

Incubations were set up in cylindrical containers with dimensions of 19.0 cm in diameter and 45.0 cm in height at a room temperature of 25 °C. Two parallel treatments were conducted in this study. In treatment A, the CW substrate material was 1:1 (volume/volume) mixtures of ceramsite and wetland soil. In treatment B, only wetland soil was used as substrate. For each treatment, the container was first filled with stones (diameters of about 3–5 mm) to a height of 5 cm. Then different substrate materials were added to a height of 37 cm in treatments A and B. Tap water was injected from an inflow port located 3 cm above the base to saturate the substrate materials. *Bruguiera gymnorhiza* were grown in each microcosm, and the plant biomass before and after incubation was listed in Table S2. The design of the microcosm was illustrated in Figure 1, and each treatment underwent three simultaneous replications.

Local domestic wastewater (filtered) was then continuously pumped into these microcosms with the inflow rate of 1 L/d to achieve a hydraulic retention time (HRT) of 6.4-day until the total N (TN) and total organic carbon (TOC) contents were stabilized in outflow (~20 days). Subsequently, the performance of wastewater treatment under three different HRT conditions were monitored in this study: 6.4-day for 21 days, 3.5-day (the inflow rate of 1.83 L/d) for 21 days, and then 1.5-day (the inflow rate of 4.27 L/d) for 20 days, which would help to understand the potential wastewater treatment capacities of this CW system (more wastewater treatment under less treatment time). Every time after adjusting HRT, the systems were first operated stably for 5 days before beginning to sample. Subsamples were taken daily from the sampling outlets for both the inflow and the outflow (40 cm above the bottom). Ammonium (NH_4_^+^) and nitrate (NO_3_^−^) of the water samples were analyzed by Ion Chromatography (IC3000). TOC and TN were detected by TOC analyzer (TOC-5000A, Shimadzu, Kyoto, Japan), while total P (TP) was measured using the NH_4_^+^ molybdate spectrophotometric method.

After a total of 71 days incubation, soil samples were collected at three sampling points including bottom (B, 8 cm above bottom), middle (M, 28 cm above bottom) and top (T, 34 cm above bottom) of CWs. Triplicate soil samples were taken from each point for further molecular analyses. 

### 2.3. DNA Extraction and Hiseq Sequencing

DNA was extracted from each soil sample (detailed information is listed in Supplementary Materials) and then sequenced. Primer set 515F-806R, which encodes the V4 region of bacterial 16S rRNA genes, was utilized for high-through sequencing by Caporaso et al. [23]. The detailed PCR program used is described in a previous study by Huang and Jaffe [24]. All sequencing was done on an Illumina Hiseq platform at Novogene Co., Beijing, China. The sequences have been deposited in the NCBI database under the accession number PRJNA632982.

The clean sequences were clustered into operational taxonomic units (OTUs) with a 97% similarity cut-off [25]. The taxonomic annotations of sequencing data were determined at the 80% threshold with the Silva SSUrRNA database.

### 2.4. Statistical Analyses

The removal rate of each pollutant was calculated according to the formula.

(1) $$\mathrm{Removal}\;\mathrm{rate}=\frac{\left(P_{i}-P_{e}\right)}{P_{i}}\times 100\%$$

where *P_i_* = pollutant concentration in influent, *P_e_* = pollutant concentration in effluent.

The results were averaged and standard errors were calculated.

Alpha-diversity indexes were calculated to evaluate the richness (Shannon and Simpson) and diversity (Chao1 and Ace) of the microbial community in each soil sample. One-way analysis of similarities (ANOSIM) was calculated to address the significance of the microbial community between different treatments. Beta-diversity matrix based on weight_unifrac was shown by principal coordinate analysis (PCoA) to illustrate the differences of microbial diversity between CWs with treatment A and B. A linear discriminant analysis effect size (LEfSe) method based on the Kruskal–Wallis test was used to identify the microbial communities that had significant differences between two different treatment groups. The LDA threshold score was set as 4.0 [26]. Functional annotation was performed using the functional annotation program of prokaryotic taxa (FAPROTAX) [27], based on the Kyoto Encyclopedia of Genes and Genomes (KEGG) database. Principle component analysis (PCA) was used to choose the principle factors for functional groups. Spearman correlation was calculated to evaluate the relationship of key pollutants and the microbial community. A significant α of 0.05 were accepted at all statistical analyses.

## 3. Results

### 3.1. Overall Performance of Wetland Microcosms with Different Treatments

**Figure 2:**
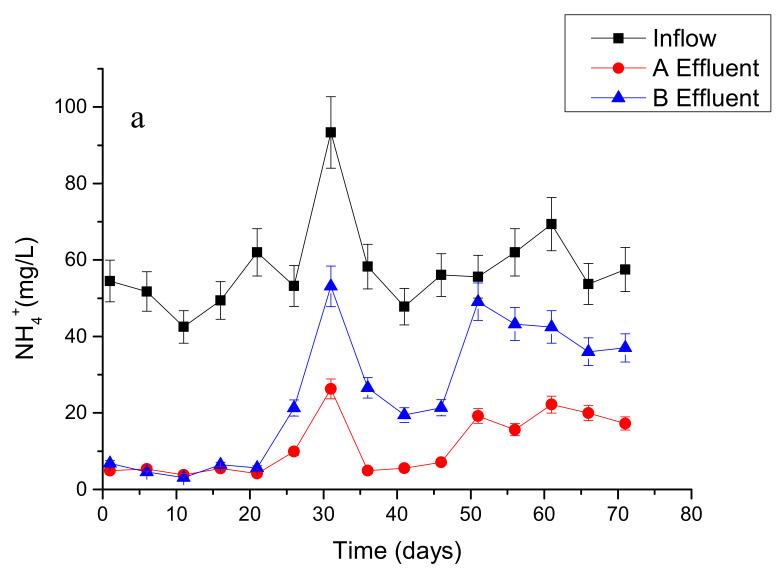

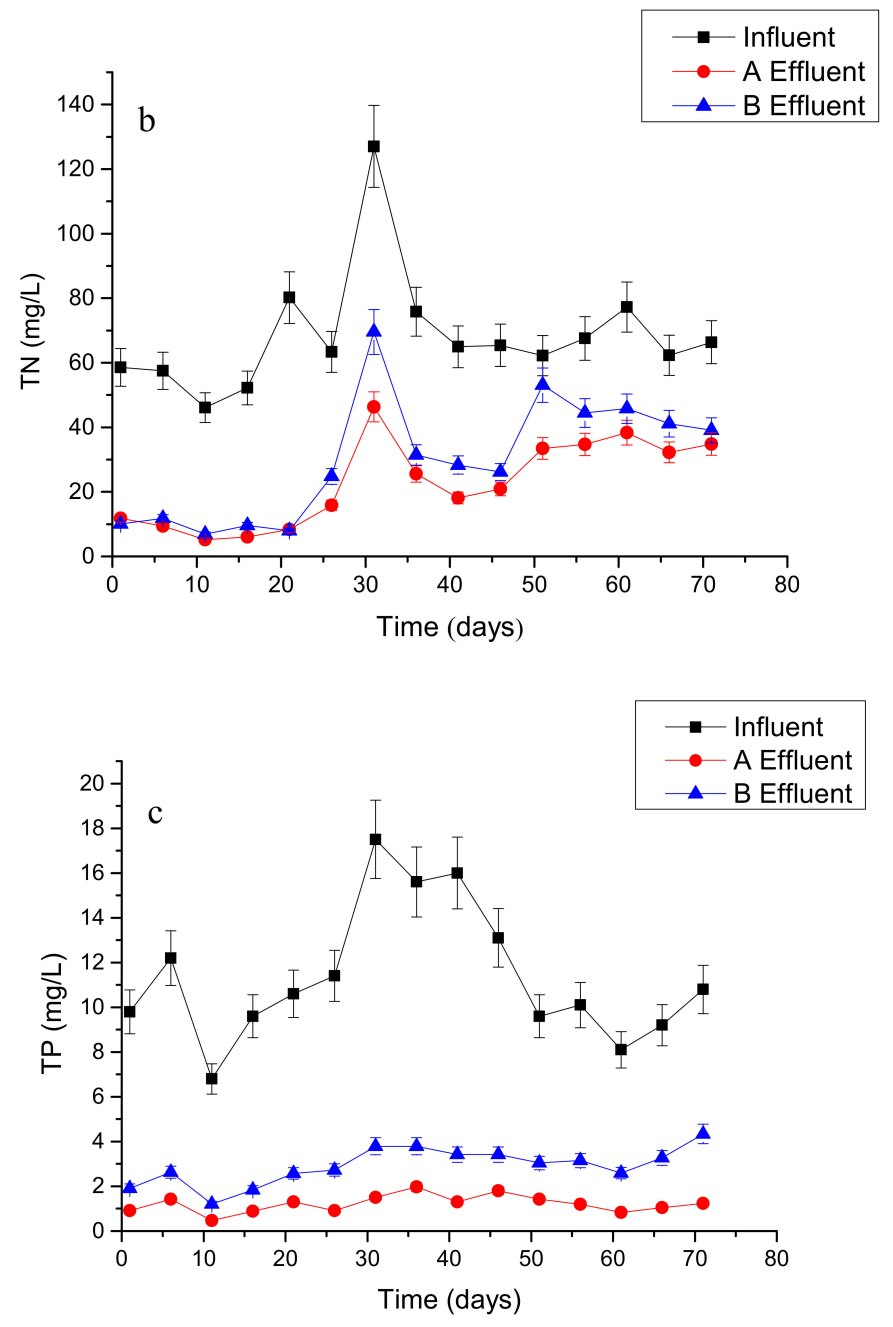
The concentrations of ammonium (NH_4_^+^) (**a**), total nitrogen (TN) (**b**) and total phosphate (TP) (**c**) from influent and effluent during 71 incubation days. Constructed wetlands using the mixture of ceramsite and wetland soil were marked as treatment A, and constructed wetlands with only wetland soils without ceramsite were labeled as treatment B.

During the operation, the inflow had 40–90 mg L^−1^ of NH_4_^+^, 40–130 mg L^−1^ of TN, and 5–18 mg L^−1^ of TP. In general, the wastewater treatment performance in the CW system of treatment A (with ceramsite) was better than that of treatment B (without ceramsite), especially under short HRT conditions (Figure 2). The average TOC removal of treatment A and of treatment B for 71 days were 59% and 38%, respectively. There was no difference in TOC removal efficiency when changing HRT during the operation. The average removal rates in treatment A for TN with HRT as 6.4-day, 3.5-day, and 1.5-day were 85%, 70%, and 50%, respectively, and for NH_4_^+^ removal were 90%, 90%, and 70%. Under the same conditions, the average removal of TN in treatment B were 80%, 60%, and 37% with HRT as 6.4-day, 3.5-day, and 1.5-day, respectively, and 90%, 60% and 35% for NH_4_^+^ removal. The treatment of TP was not affected by HRT, while the efficiency was also higher in the CW system of treatment A than treatment B.

### 3.2. Microbial Diversity and Composition in Wetland Microcosms

In total, 80,057–80,303 clean reads were obtained for these soil samples, which were then clustered into 2857–5281 OTUs. Rarefaction analysis (Figure S1) and Good’s coverage of 0.979–0.988 indicated that the microbial communities in all soil samples could be covered by the depth of high-through sequencing. Alpha diversity indexes of community richness and community diversity were compared between treatment A and treatment B (Table S2). Shannon indexes in treatment A were higher than those in treatment B, and were also higher in the bottom and middle samples of each microcosm than the top ones. All these indexes suggested that microbial diversity was higher in the microcosms with ceramsite treatment than those with only soil as the substrate material.

**Figure 3:**
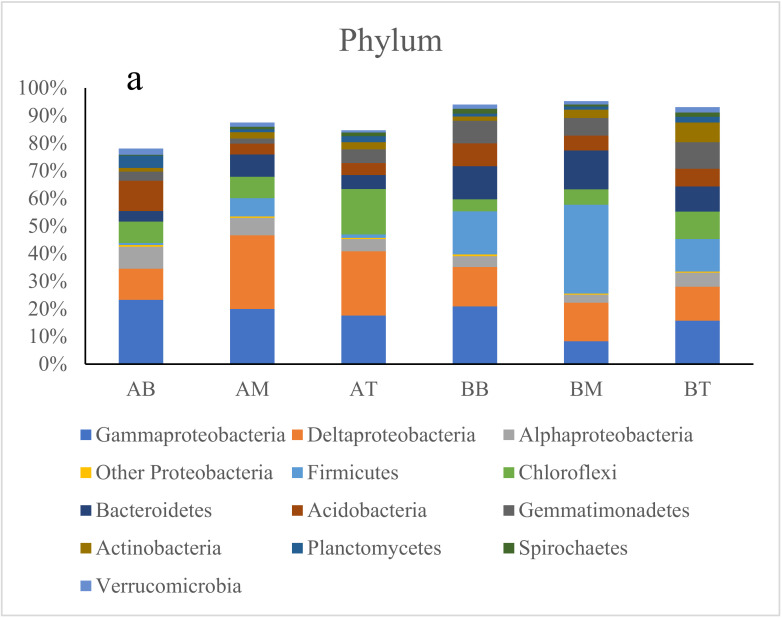

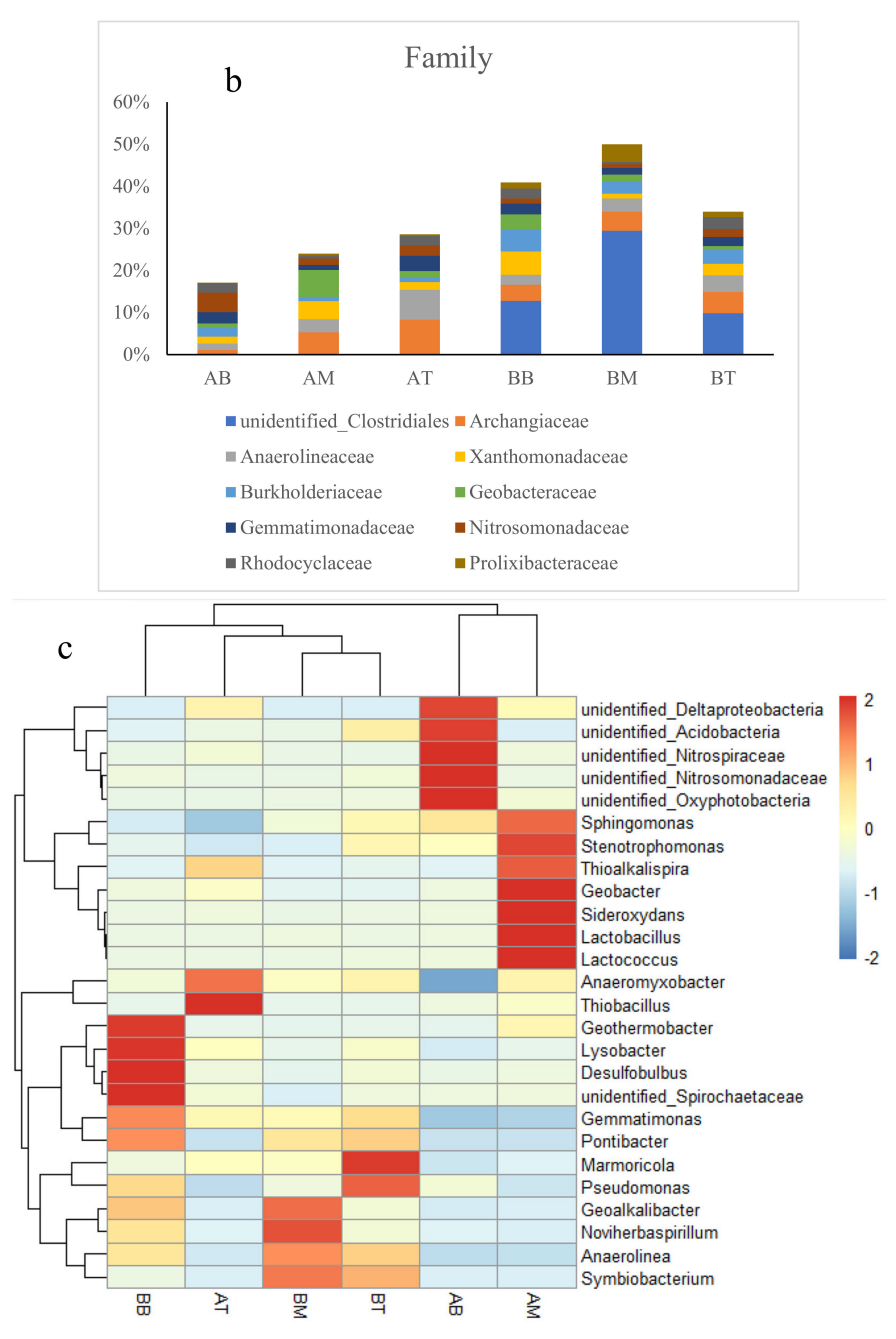
The dominant microbial community. (**a**) Top 10 phyla (Proteobacteria was listed to the class level); (**b**) top 10 families; and (**c**) heatmap of genera of at least one sample >1%. AB: bottom of treatment A; AM: middle of treatment A; TP: top of treatment A; BB: bottom of treatment B; BM: middle of treatment B; BP: top of treatment B.

The top 10 phyla in these CWs are illustrated in Figure 3a, while *Proteobacteria* was classified to the class level. In all cases, *Proteobacteria*, particularly *Gammaproteobacteria* and *Deltaproteobacteria*, dominated in all soil samples with 25.5–53.4%. *Firmicutes* was the second most abundant microorganism in CWs with treatment B (11.9–32.2%), followed by *Bacteroidetes* (9.1–14.1%). In contrast, the top two dominant microorganisms in CWs with treatment A were *Chloroflexi* (7.7–16.4%) and *Acidobacteria* (3.9–11.0%).

In terms of family level, the top three families in CWs with treatment A were *Archangiaceae* (4.7%), *Anaerolineaceae* (3.9%) and *Geobacteraceae* (2.9%), while *Clostridiales* were dominated in CWs with treatment B (17.3%) (Figure 3b).

At the genus level, the top 26 dominant genera (higher than 1% in total) are shown in Figure 3c. The dominant genera between the two treatments were different after incubation. *Anaeromyxobacter*, *Geobacter*, *Stenotrophomonas*, *Thiobacillus*, *Sphingomonas*, *Thioalkalispira*, and *Acidobacteria* were dominated in treatment A, and accounted for up to 13.2%. In samples from treatment B, *Anaeromyxobacter*, *Symbiobacterium*, *Lysobacter*, *Pseudomonas*, *Pontibacter*, *Marmoricola*, *Gemmatimonas* and *Anaerolinea* were much higher.

**Figure 4:**
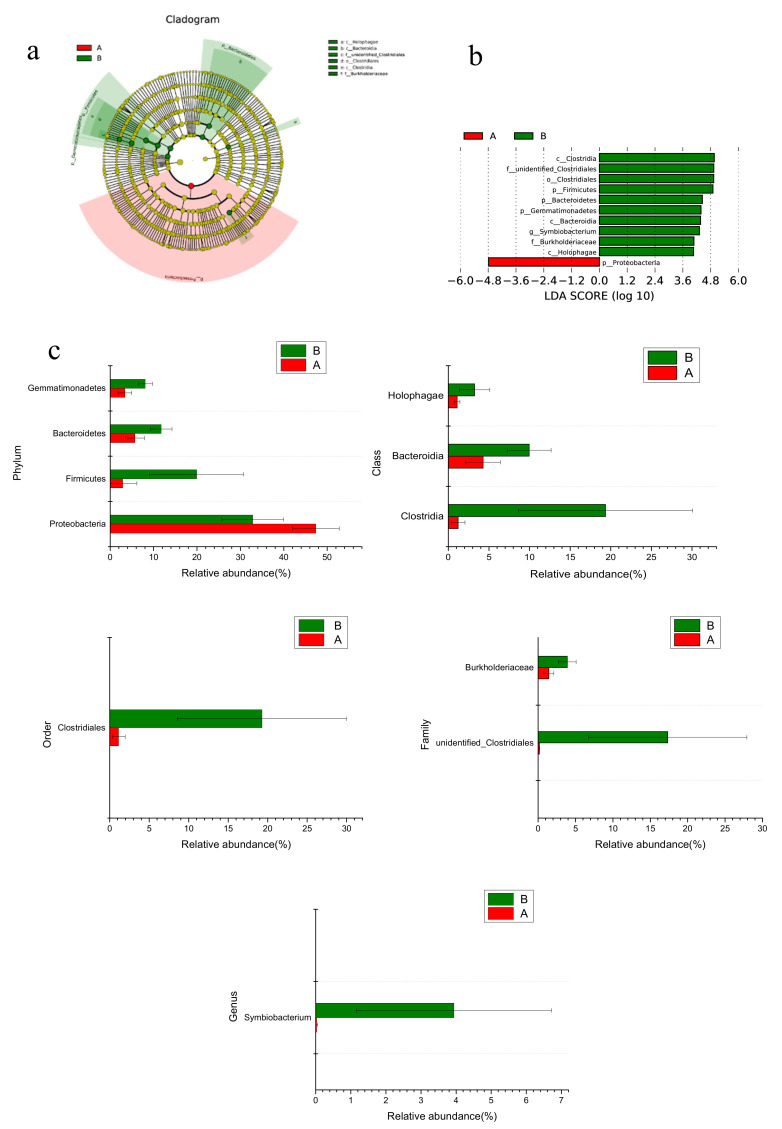
LEfSe analysis of microbial abundance between treatment A with ceramsite and treatment B without ceramsite. (**a**) is the cladogram of microbial communities. (**b**) is the LDA score identified for the size of differentiation with a threshold value of 4.0. (**c**) includes comparisons of microbial abundances with significant differences from phylum to genus level.

ANOSIM results indicated no significant differences in the structures of the microbial communities (*p*  > 0.05) between the two CWs treatments. The microbial abundance showed significant differences between treatment A and treatment B via the LEfSe method (Figure 4). Under a LDA threshold of 4.0, groups presented significant differences in different systems, including, 4 groups at the phylum level, *Preoteobacteria*, *Firmicutes*, *Bacteroidetes, and Gemmatimonadetes*; 3 groups at the class level, *Clostridia*, *Bacteroidia*, and *Holophagae*; 1 group at the order level, *Clostridiales*; 2 groups at the family level, *unindentified Clostridiales* and *Burkholderiaceae*; and 1 group at the genus level, *Symbiobacterium*.

**Figure 5:**
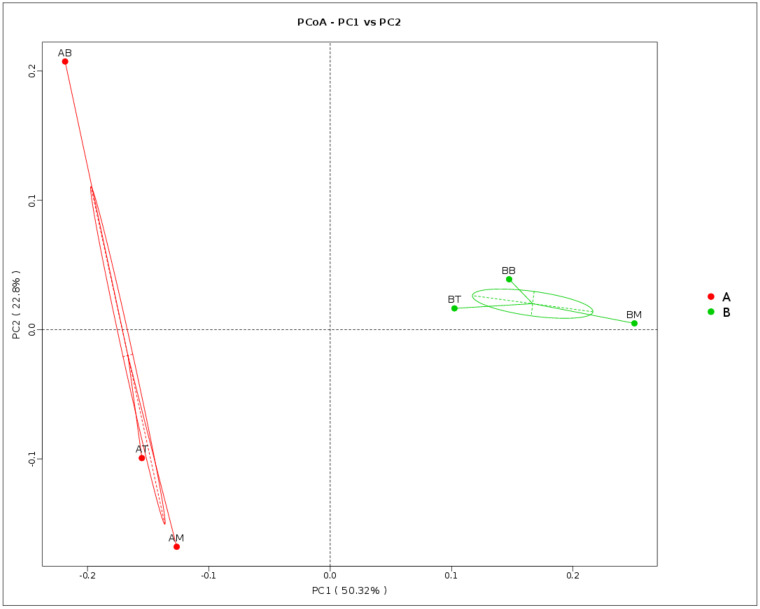
Principle Coordinate analysis of microbial composition in soil samples, which suggested the microbial community structure in treatment B (without ceramsite) was similar, while the microbial community in treatment A (with ceramsite) differed over depth.

The PCoA figure also illustrated that the microbial diversity was different between CWs with treatment A and treatment B. The points shown for treatment A were spread fairly far apart while those for treatment B were clustered, which suggested that the microbial community structure in all layers of treatment B were similar (Figure 5).

### 3.3. Functional Prediction

**Figure 6:**
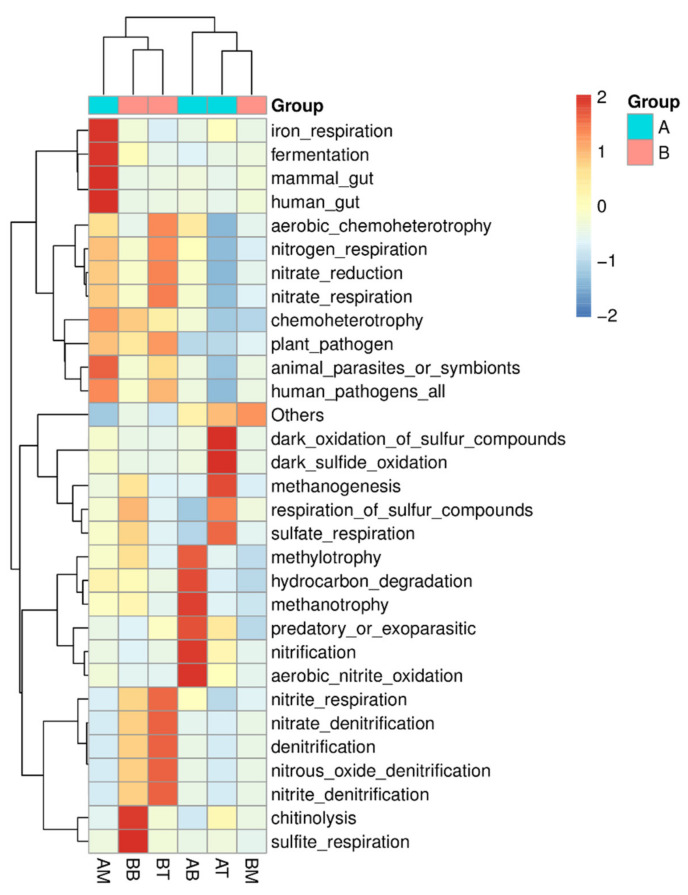
Heatmap of predicted functions based on the program of functional annotation of prokaryotic taxa (FAPROTAX). Z-score was used to illustrate the relationship among samples.

**Figure 7:**
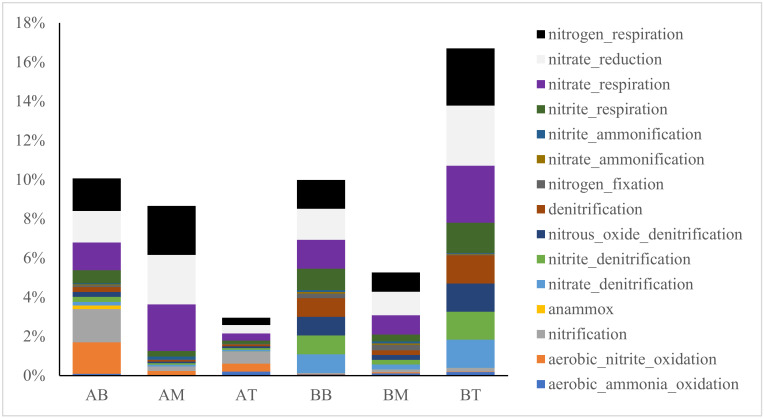
The percentage of genera involved in biogeochemical cycles of nitrogen. The genera were grouped into specific metabolic pathways based on Kyoto Encyclopedia of Genes and Genomes (KEGG) database, then the percentage of each pathway was counted as the total percentage of genera involved in this pathway.

Microbial functional predictions based on the KEGG database were conducted to study the functions of the microorganisms in the CWs (Figure 6 and Figure 7). The dominant functions in microcosm with treatment A were fermentation, iron respiration, S oxidation, methane production and oxidation, while treatment B was dominated by functional groups involved in sulfide oxidation.

In terms of N functions, both Figure 6 and Figure 7 revealed that microcosms with treatment A had more nitrification than those with treatment B after the 71 days operation, particularly in the bottom layers. Nitrification in the bottom of microcosm with treatment A were 1.61% and 1.70%, while those in B were 0.02% and 0.06%, respectively. Anammox was only found in the bottom layer of samples from treatment A (0.18%). Meanwhile, the functions related to denitrification was less in treatment A samples than samples from CWs with treatment B, particularly in the top layer (0.09% in treatment A compared with 1.43% in treatment B).

### 3.4. Correlation of Pollutant Concentrations and Microbial Components

The Spearman correlation was established for pollutant concentrations vs. microbial abundance at class or genus levels (Table 1). Two classes, *Anaerolineae* and *unidentified_Actinobacteria*, showed a significant negative relationship with NH_4_^+^, TN and TP, while *Gammaproteobacteria* and *Holophagae* had a strong positive relationship with pollutants. At the genus level, *Anaeromyxobacter* displayed a significant negative correlation with all key pollutants, and *Marmoricola* had a significant negative correlation to only TP.

## 4. Discussion

### 4.1. Ceramsite Substrate Enhanced Wastewater Treatment Efficiency in CWs

The pollutant removal efficiency in CWs using the mixture of ceramsite and wetland soil was significantly better than that in CWs without ceramsite, and this result was more obvious under a shorter HRT condition (Figure 2). No significant difference was found in the removal rates of all pollutants in CWs with treatment A and treatment B under a longer HRT condition. Although a shorter HRT also lowered the wastewater treatment efficiency, CWs with treatment A could maintain pollutant removal rates at 50%, 70%, and 88% for TN, NH_4_^+^ and TP, respectively, with a HRT of 1.5-day, which were 1.35, 2 and 1.31 times as in treatment B (Figure 2). This result was consistent with other studies that showed by using ceramsite type material as wastewater fillers it could enhance treatment efficiency [21,28,29]. On the one hand, the porous properties of ceramsite could provide a more aerobic condition to stimulate biological oxidation of C and N substrates; on the other hand, the high specific surface area and high water absorption character could maintain sufficient substrate for the microbe to conduct pollutant biodegradation. In particular, the removal of P in CWs with ceramsite was higher due to not only more space for P cyclers but also the better P binding capacity in the ceramsite-type materials [30,31].

### 4.2. Changes of Microbial Community in CWs During Operation

After 71 d incubation, the microbial community structures from two treatments were different (Figure 3 and Figure 4). Data showed that NH_4_^+^ oxidizers, *Nitrosomonas* and *Nitrosomonadaceae* [1], in treatment A was four times higher than treatment B. S oxidizers *Thiobacillus* and *Thioalkalispira* [14,32], were about 2.66% and 2.29% in microcosms with treatment A, while their abundance was less than 0.02% in microcosm with treatment B. *Geobacter*, a well-known iron reducer [15], was more abundant in treatment A than treatment B. *Candidatus Competibacter*, a microorganism responsible for P removal, was only found in CWs with treatment A [16]. The relationship of pollutants and these key microbial components indicated that these microbes could contribute to pollutant removals. All these results showed that ceramsite material can stimulate key microbes for domestic wastewater treatment.

It is interesting to find out that ceramsite substrate not only stimulated the functional microbial groups, but also affected their spatial habitat in these vertical flow systems. According to PCoA (Figure 5), microbial components from different layers were different in treatment A. Data showed NH_4_^+^ oxidizers had more abundance in the bottom (1.79%) than the middle (0.13%) and top (0.09%) of the microcosm. A higher *Geobacter* proportion in the middle and top layers was observed as well, which suggested that the shift of redox potential went from bottom to top in the microcosm. In vertical flow systems, the microbial degradation efficiencies may be inhibited by a lack of aeration [6]. Our results implied that ceramsite substrates may improve the wastewater treatment efficiencies by enhancing the microbial degradation in the bottom of vertical flow systems. However, this was not found in only soil substrates.

### 4.3. Ceramsite Substrate Enhanced Nitrification in CWs

As a main pollutant in domestic wastewater, the mechanism of N removal and its contributors in CW systems with ceramsite substrate was highlighted here. Functional predictions (Figure 6 and Figure 7) suggested that during operation, the proportion of nitrifiers increased in CWs with treatment A, thus contributing to potential enhanced nitrification. Since NH_4_^+^ and NO_2_^−^ oxidation are both the rate-limited process in N cycle, increasing their efficiency will greatly improve N removal in wastewater treatment. Archaeal NH_4_^+^ oxidizers (AOA) [33,34] also showed a similar trend as AOB in the CWs with treatment A, and had 0.88% in the bottom samples. Anammox bacteria, *Candidatus Brocadia* [13,35], was observed in the bottom of CWs with treatment A, also supported the idea that the oxidation of NH_4_^+^ in this system had been improved. Newly discovered NH_4_^+^ oxidizers such as Feammox (NH_4_^+^ oxidizer coupled with iron reduction) bacteria [24] and comammox (complete nitrification) bacteria [36,37] were not observed in this study. NO_2_^−^ oxidizers, including *Nitrospinae*, *Nitrospiraceae* and *Nitrospirae* [38,39] were all found in higher concentrations in CWs with treatment A than those with treatment B, and also in higher concentrations in the bottom of CWs with treatment A as opposed to the middle and top layers. These results explained that nitrification was enhanced in treatment A, particularly in the bottom layers. In contrast, the relative number of denitrifiers was reduced in CWs with treatment A (average of 0.14% in treatment A and 0.88% in treatment B). Two well-known denitrifiers, *Burkholeriacea* and *Pseudomona* [12,40], had significantly lower percentages in sample from treatment A than those from treatment B (Figure 7).

## 5. Conclusions

Compared to the use of soil as the substrate, using ceramsite as the substrate enhanced the removal of pollutants from CW systems, particularly under a short HRT condition. The significant difference in microbial communities between CW systems with and without ceramsite showed that ceramsite could help to enhance the percentage of beneficial microorganisms (N, S, P) for wastewater treatment, particularly in the bottom layers. More specifically, the CW with ceramsite system enhanced N removal rate because it increased the efficiency and abundance of nitrifiers.

## Figures and Tables

**Table 1:** Spearman correlation of key pollutants and microbial components (top 10) at the class and genus levels. TN: total nitrogen; TP: total phosphate. * *p* < 0.05; ** *p* < 0.01. *n* = 3.

	NH_4_^+^	TN	TP
**Class Level**			
***Clostridia***	0.145	0.145	0.000
***Deltaproteobacteria***	−0.406	−0.406	−0.239
***Gammaproteobacteria***	0.580	0.580	0.717
***Anaerolineae***	−0.986 **	−0.986 **	−0.956 **
***Bacteroidia***	0.145	0.145	0.000
***Alphaproteobacteria***	0.029	0.029	0.120
***Holophagae***	0.638	0.638	0.478
***unidentified_Actinobacteria***	−0.870 *	−0.870 *	−0.956 **
***Bacilli***	0.058	0.058	0.000
***unidentified_Gemmatimonadetes***	−0.058	−0.058	0.000
**Genus**			
***Anaeromyxobacter***	−0.928 **	−0.928 **	−0.837 *
***Symbiobacterium***	0.232	0.232	0.000
***Geobacter***	−0.116	−0.116	0.120
***Lysobacter***	−0.232	−0.232	−0.239
***Stenotrophomonas***	0.116	0.116	0.120
***Thiobacillus***	−0.294	−0.294	−0.061
***Marmoricola***	−0.725	−0.725	−0.837 *
***Pontibacter***	0.000	0.000	−0.120
***Thioalkalispira***	−0.691	−0.691	−0.546
***Pseudomonas***	0.406	0.406	0.239

## Supplementary Materials

The following are available online at https://www.mdpi.com/1660-4601/17/13/4692/s1; Table S1 Leaching concentration of heavy metals in ceramsite (mg/L); Table S2 Plant biomass before and after incubation; Table S3 Community diversity indices (Shannon and Simpson), estimated community richness indices (Chao1 and Ace) and coverage of the 16S rRNA in soil samples; Figure S1 Rarefaction curve of OTUs and 16S rRNA sequencing number; Figure S2 Principle Component analysis of predicted functions in soil samples.
