# Supplementary material for: Ceramsite Facilitated Microbial Degradation of Pollutants in Domestic Wastewater
Source: Int J Environ Res Public Health. 2020 Jun 30;17(13):4692. doi: 10.3390/ijerph17134692 (PMC7369936; doi:10.3390/ijerph17134692)
Supplement: Supplementary file 1 [file ijerph-17-04692-s001.docx]

**Supplementary Material**

Ceramsite Facilitated Microbial Degradation of Pollutants in Domestic Wastewater

Qiong Wan ^1^, Qingji Han ^2^, Hailin Luo ^3^, Tao He ^3^, Feng Xue ^4^, Zihuizhong Ye ^5^, Chen Chen ^3,^* and Shan Huang ^6^

^1^ School of Architecture and Civil Engineering, Xi’an University of Science and Technology, Xi’an 710054, China; wq6675@xust.edu.cn

^2^ Xi’an Research and Design Institute of Wall & Roof Materials Co., Ltd., Xi’an 710061, China; hanqingjih@163.com

^3^ State Environmental Protection Key Laboratory of Urban Ecological Environment Simulation and Protection, South China Institute of Environmental Sciences, Ministry of Ecology and Environment of China, Guangzhou 510535, China; luohailin@scies.org (H.L.); hetao@scies.org (T.H.)

^4^ Xi’an Pengyi Environmental Engineering co. Ltd., Xi’an 710054, China; melon75@163.com

^5^ Stuart Country Day School, Princeton, NJ 08540, USA; zye_22@stuartschool.org

^6^ Department of Civil and Environmental Engineering, Princeton University, Princeton, NJ 08544, USA; shanh@princeton.edu

***** Correspondence: chenchen7@scies.org; Tel.: +86-20-29119810

Detail description of DNA extraction and sequencing

DNA Extraction

DNA was extracted from each soil samples, as described in the manual of the Fast DNA spin kit (Bio 101, Qbiogene Inc., Carlsbad, CA, USA). The concentration and purity of the isolated total DNA was checked using a NanoDrop 2000 Spectrophotometer (Thermo Fisher Scientific, Marietta, OH, USA), and its integrity was evaluated by electrophoresis on ethidium bromide-stained agarose gels (Thermo Scientific 2X RNA Loading Dye).

Illumina HiSeq Sequencing and data processing

To further study the changes in the bacterial communities, sequencing of 16S rRNA gene and functional genes involved in nitrification or denitrification were performed on an Illumina HiSeq platform. The V4 region of the 16S rRNA gene of bacteria were amplified using primer-set 515F-806R following methods suggested by Caporaso et al. (Caporaso et al., 2012). All PCR reactions were carried out with a Phusion^®^ High-Fidelity PCR Master Mix (New England Biolabs, Ipswich, MA, USA), and the PCR products were quantified and purified before sequencing. Sequencing libraries were generated using TruSeq^®^ DNA PCR-Free Sample Preparation Kit (Illumina, San Diego, CA, USA) following the manufacturer’s recommendations and index codes were added. The library quality was assessed on the Qubit@ 2.0 Fluorometer (Thermo Scientific, Waltham, MA, USA) and Agilent Bioanalyzer 2100 system. Finally, the library was sequenced on an Illumina MiSeq platform and 250~500 bp paired-end reads were generated. All amplicon sequencing was conducted on an Illumina HiSeq platform at Novogene co., Beijing, China.

Operational taxonomic units (OTUs) were clustered with a 97% similarity cut-off using the Uparse software (Edgar, 2013). A representative sequence for each OTU was screened for further annotation. The taxonomy of each 16S rRNA gene sequence was analyzed with Muther (version v.1.30.1) against the Silva SSUrRNA database using a confidence threshold of 0.8–1. Amplicon sequences are available through NCBI under the BioProject ID PRJNA632982.

**Table S1.** Leaching concentration of heavy metals in ceramsite (mg/L). –, not detected.

|  | **Barium** | **Arsenic** | **Zinc** | **Nickle** | **Lead** | **Chromium** | **Copper** | **Cadium** |
| --- | --- | --- | --- | --- | --- | --- | --- | --- |
| **Leaching concentration** | 0.908 | - | - | 0.685 | 1.680 | 0.434 | 0.160 | 0.084 |
| **Quality control** | 100 | 5 | 100 | 5 | 5 | 15 | 100 | 1 |

**Table S2.** Plant biomass before and after incubation. Treatment A: with ceramsite as substrate; treatment B: without ceramsite as substrate.

| **Treatment** | **Before Incubation (g)** | **After Incubation (g)** |
| --- | --- | --- |
| A | 31.1 ± 3.7 | 31.6 ± 3.9 |
| B | 30.8 ± 2.0 | 30.3 ± 2.0 |

**Table S3.** Community diversity indices (Shannon and Simpson), estimated community richness indices (Chao1 and Ace) and coverage of the 16S rRNA in soil samples.

| **Sample Name** | **Shannon** | **Simpson** | **Chao1** | **ACE** | **Coverage** |
| --- | --- | --- | --- | --- | --- |
| AB | 10.532 | 0.998 | 5158 | 5362 | 0.979 |
| AM | 9.272 | 0.992 | 4426 | 4698 | 0.981 |
| AP | 9.620 | 0.993 | 4521 | 4786 | 0.980 |
| BB | 8.667 | 0.992 | 3068 | 3270 | 0.986 |
| BM | 7.750 | 0.974 | 2674 | 2879 | 0.988 |
| BP | 9.257 | 0.994 | 3598 | 3754 | 0.985 |


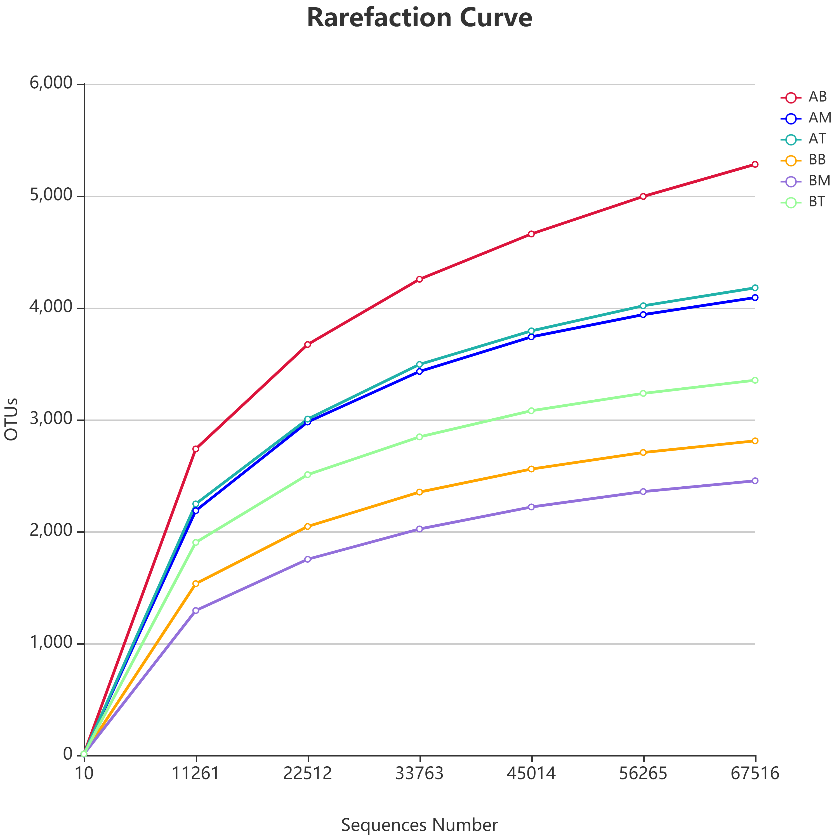


**Figure S1.** Rarefaction curve of OTUs and 16S rRNA sequencing number.


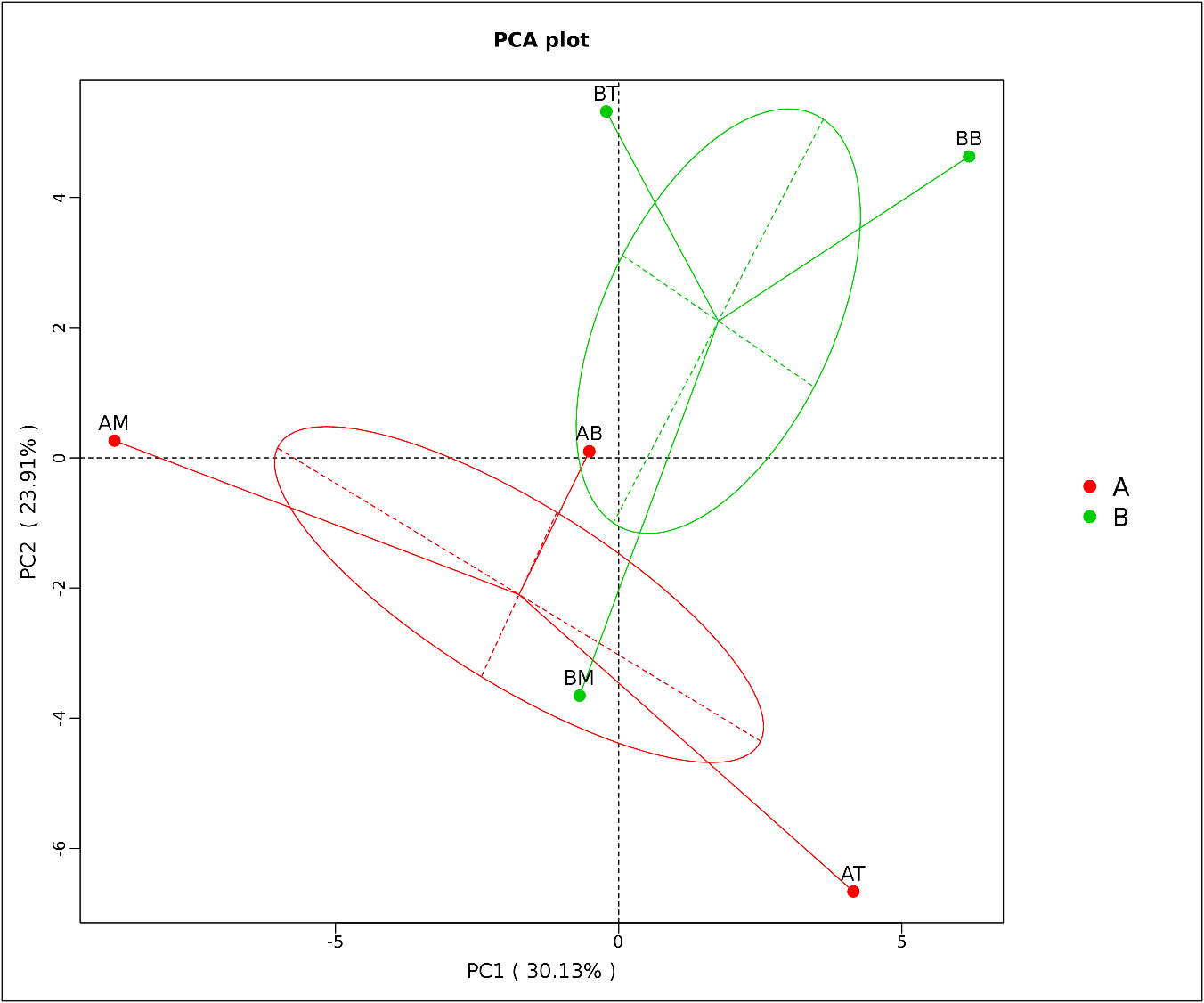


**Figure S2.** Principle Component analysis of predicted functions in soil samples.
